# The homeostatic set point of the hypothalamus-pituitary-thyroid axis – maximum curvature theory for personalized euthyroid targets

**DOI:** 10.1186/1742-4682-11-35

**Published:** 2014-08-08

**Authors:** Melvin Khee-Shing Leow, Simon L Goede

**Affiliations:** 1 Department of Endocrinology, Tan Tock Seng Hospital, 11 Jalan Tan Tock Seng, Singapore 308433, Republic of Singapore; 2 Clinical Research and Innovation Office, Tan Tock Seng Hospital, 11 Jalan Tan Tock Seng, Singapore 308433, Republic of Singapore; 3 Department of Medicine, National University Hospital, 5 Lower Kent Ridge Road, Singapore 119074, Republic of Singapore; 4 Office of Clinical Sciences, Duke-NUS Graduate Medical School, 20 College Road, Singapore 169856, Republic of Singapore; 5 Brenner Centre for Molecular Medicine, Singapore Institute for Clinical Sciences, 30 Medical Drive, Singapore 117609, Republic of Singapore; 6 Yong Loo Lin School of Medicine, 10 Medical Drive, Singapore 117597, Republic of Singapore; 7 Clinical Trials & Research Unit, Changi General Hospital, 2 Simei Street 3, Singapore 529889, Republic of Singapore; 8 Electronic Engineering, Oterlekerweg 4, 1841 GP Stompetoren, The Netherlands

**Keywords:** Hypothalamus-pituitary-thyroid axis, Log-linear, Feedback, Personalized medicine, Set point, Loop gain

## Abstract

**Background:**

Despite rendering serum free thyroxine (FT4) and thyrotropin (TSH) within the normal population ranges broadly defined as euthyroidism, many patients being treated for hyperthyroidism and hypothyroidism persistently experience subnormal well-being discordant from their pre-disease healthy euthyroid state. This suggests that intra-individual physiological optimal ranges are narrower than laboratory-quoted normal ranges and implies the existence of a homeostatic set point encoded in the hypothalamic-pituitary-thyroid (HPT) axis that is unique to every individual.

**Methods:**

We have previously shown that the dose–response characteristic of the hypothalamic-pituitary (HP) unit to circulating thyroid hormone levels follows a negative exponential curve. This led to the discovery that the normal reference intervals of TSH and FT4 fall within the ‘knee’ region of this curve where the maximum curvature of the exponential HP characteristic occurs. Based on this observation, we develop the theoretical framework localizing the position of euthyroid homeostasis over the point of maximum curvature of the HP characteristic.

**Results:**

The euthyroid set points of patients with primary hypothyroidism and hyperthyroidism can be readily derived from their calculated HP curve parameters using the parsimonious mathematical model above. It can be shown that every individual has a euthyroid set point that is unique and often different from other individuals.

**Conclusions:**

In this treatise, we provide evidence supporting a set point-based approach in tailoring euthyroid targets. Rendering FT4 and TSH within the laboratory normal ranges can be clinically suboptimal if these hormone levels are distant from the individualized euthyroid homeostatic set point. This mathematical technique permits the euthyroid set point to be realistically computed using an algorithm readily implementable for computer-aided calculations to facilitate precise targeted dosing of patients in this modern era of personalized medicine.

## Introduction

### The enigma of symptomatic ‘euthyroidism’

Thyroid stimulating hormone [TSH] and free thyroxine [FT4] constitute most standard thyroid function tests (TFT) and play a central role in the process of diagnosis and treatment of thyroid diseases such as hypothyroidism and thyrotoxicosis. Evidence indicates that the laboratory-quoted [TSH] reference range is not always appropriate for everyone in clinical practice [1-5]. Clinicians recognize a common phenomenon of patients who persistently experience residual symptoms of thyrotoxicosis or hypothyroidism despite having achieved circulating levels of [FT4] and [TSH] within the normal ranges [4,6-8]. Hence, achieving a [TSH] within a healthy range may still depart significantly from the true euthyroid set point of any given individual [4].

To solve this problem, it is crucial to appreciate that the normal operation of a negative feedback loop implies the existence of a point of reference, or a set point intrinsic to the system [9-12]. The HP unit acts as a sensitive metabolic sensor and master controller for regulating the thyroid output to a very narrow physiological ‘set range’ (termed the homeostatic set point) appropriate to the individual at euthyroidism. Any departure of [FT4] and [TSH] away from this set point leads to a disequilibrium that influences the HP unit to alter its response so as to restore the system back towards the set point [13]. In this servo-control system, the set point in an intact hypothalamus-pituitary-thyroid (HPT) axis of a healthy euthyroid individual represents the ideal ‘personalized’ TFT target that results in an optimal healthy state.

In physiology, both optimal control and steady states are critical for survival and good health. Homeostatic mechanisms operate with great efficiency to restore equilibrium from acute perturbations. However, various biological constraints due to disease or environment changes may lead to failure of achievement of the steady state and physiological optimum. In the terminology of systems theory, a system in steady state has numerous characteristics that are constant over time such that the partial derivative of these characteristics with respect to time is zero and the recently observed behavior of the system will continue into the future. The steady state is usually not attained till sometime after an initial ‘transient warm up’ period. Notably, the steady state is more general than dynamic equilibrium in that the former may not be a state of dynamic equilibrium as some processes are irreversible while the latter is always a steady state in which the forward and reverse processes are occurring at the same rate. Optimal feedback control refers to the process of determining the control and state trajectories for a dynamic system over time to maximize or minimize a performance index while satisfying specified constraints where the control variables are determined as functions of the current state of the system, a principle widely exploited as part of Nature’s strategies to confer the best probabilities for survival.

For the HPT axis, optimal feedback control and systems theory explain how thyrotropic hormones are governed and maintained within a preferential normal range that allows people to live and enjoy good health. In individuals whose HPT feedback loops have been disrupted by diseases, we hypothesize that their state of health may be restored back to the original, provided that their [FT4] and [TSH] are driven towards their pre-disease set points. Molecular, tissue, biochemical and clinical euthyroidism appropriate to the individual, will ultimately be achieved when the [FT4] and [TSH] are both therapeutically constrained to dwell within the set point and its immediate vicinity over a sufficiently long timeframe.

### Inverse exponential law and the logarithmic-linear function

Mathematical modeling of the HP function had previously yielded an inverse exponential law governing the [TSH] and [FT4] relationship which is sigmoidal in profile with an inflexion point that can be approximated by a negative exponential curve in most practical instances [14]. This fits the well established observation of an approximately log-linear function between [TSH] and [FT4] that to date remains the best biophysical first principle coupling these two hormones’ concentrations [15-21]. Hence, we derived an individualized [FT4] – [TSH] relationship (hereby termed the hypothalamic-pituitary (HP) function) using a minimal model based on a parameterized negative exponential model of the HPT axis with two degrees of freedom that opens up a novel approach for precise individualized dosing of levothyroxine (L-T4) substitution and anti-thyroid drug (ATD) therapy [22,23].

A key observation we discovered, is the consistent localization of the normal population ranges of [TSH] and [FT4] values within the “knee” region coinciding to the most pronounced bend on the negative exponential plot of the HP function on the TSH-FT4 Cartesian plane [22]. The obvious implication of this discovery is that the euthyroid set point must be localized somewhere over this region that optimizes the system’s control ability of the HPT axis to calibrate the [TSH] output robustly for tight homeostasis within a narrow physiological window for survival advantage. This leads us logically to conjecture that the euthyroid set point of the HPT axis should correspond to the point of maximum curvature of the HP function for optimal efficiency in homeostatic control. We evaluate this postulate from a mathematical perspective and validate it using clinical data. An independent confirmation is shown in a HPT loop gain optimization analysis according to control theory principles [13]. With these results, we devise the foundations of a novel approach to improve diagnostics and the related therapeutic strategies for hypothyroid and hyperthyroid patients.

Although triiodothyronine [T3] is the main active thyroid hormone, the response characteristics analysis as confined purely to the relationship between [FT4] and [TSH] is sufficient for all intents and purposes. This is valid because the local DIO2 (type II 5’-deiodinase) in the hypothalamus and pituitary converts [FT4] into [T3] locally, which together with ambient circulating [T3] contributed by peripheral DIO1 (type I 5’-deiodinase)-mediated deiodination of [FT4] and by direct thyroidal secretion of [T3], then exerts negative feedback signal responsible for the inhibition of [TSH]. Moreover, a model involving key observable variables of interest (primarily [FT4] and [TSH]) is pragmatic and will thus find the widest clinical application. In contrast, any modeling attempt incorporating [T3] or [FT3] to encompass a more complete picture actually suffers from the serious drawback of severely limited practical utility as both [T3] and [FT3] are generally measured only in specific clinical situations rather than routinely in the assessment and monitoring of thyroid status [6].

## Methods

### Parameterization of the negative exponential HP characteristic

As mentioned above, the properties and form of the HP characteristic have been derived from fundamental physiological considerations [14]. This theory led to our construct of two independent HP model parameters. The parameterized expression linking the concentrations of the [TSH] and [FT4] is:

(2.0.1) $$\left[\mathrm{TSH}\right]=S\exp\left(-\varphi \left[\mathrm{FT}4\right]\right)$$

This model has been published [22] and also clinically validated in another paper [23] This characteristic has been described as a 2-dimensional (*S* and *φ*) model where parameters *S* and *φ* are real positive numbers which are derived from measured individualized thyroid function tests.

One and only one exponential function is completely defined by the coordinates of two different points *P*_1_ and *P*_2_ in the [FT4]-[TSH] plane according to

(2.0.2) $$\varphi =\left(\frac{1}{\left({\left[\mathrm{FT}4\right]}_{1}-{\left[\mathrm{FT}4\right]}_{2}\right)}\right)\ln\left(\frac{{\left[\mathrm{TSH}\right]}_{2}}{{\left[\mathrm{TSH}\right]}_{1}}\right)$$

(2.0.3) $$S={\left[\mathrm{TSH}\right]}_{1}\exp\left(\varphi {\left[\mathrm{FT}4\right]}_{1}\right)={\left[\mathrm{TSH}\right]}_{2}\exp\left(\varphi {\left[\mathrm{FT}4\right]}_{2}\right)$$

(2.0.4) $$P_{1}=\left({\left[\mathrm{FT}4\right]}_{1},{\left[\mathrm{TSH}\right]}_{1}\right)\;\mathrm{and}\;P_{2}=\left({\left[\mathrm{FT}4\right]}_{2},{\left[\mathrm{TSH}\right]}_{2}\right)$$

Every exponential function has a unique identifiable point of maximum curvature from which the complete function can be reconstructed as elaborated below. Thus, the HP function can also be determined by that single specific point. The accuracy of *S* and *φ* is dependent on the fitting quality of the exponential function as discussed in detail elsewhere [22]. This relates also to the definition and qualification of possible outliers [24].

### Calculation of the set point of the HPT axis from a defined HP function

In this treatise, the set point for euthyroid homeostasis in the HPT axis feedback loop is postulated to occur in the point of the HP function where the sensitivity for any change around this point on the curve is maximal. This point of maximum curvature and thus maximal sensitivity for change can be found according standard curvature theory. The radius of curvature at a point of a function is defined as R and the curvature is defined as K. Thus,

(2.0.5) $$K=\frac{1}{R}$$

If the function to be examined is defined as y = *f* (*x*) = *f*1 then the curvature K of *y* is defined as

(2.0.6) f2=K=d2ydx21+dydx232

Using the HP function of the hypothalamic-pituitary unit, we have

(2.0.7) $$\left[\mathrm{TSH}\right]=S\exp\left(-\varphi \left[\mathrm{FT}4\right]\right)$$

Writing [TSH] = *y* and [FT4] = *x*, we have

(2.0.8) $$y=S\exp\left(-\mathrm{\varphi x}\right)$$

Working out (2.0.8) according to (2.0.6), we find

(2.0.9) K=φ2Sexp−φx1+φ2S2exp−2φx32

For the value of x where K is a maximum, the following condition will be investigated:

(2.0.10) $$\frac{\mathrm{dK}}{\mathrm{dx}}=0,$$

then

(2.0.11) $$f3=\frac{\mathrm{dK}}{\mathrm{dx}}=\frac{\varphi^{3}S\exp\left(-\mathrm{\varphi x}\right){\left\{1+\varphi^{2}S^{2}\exp\left(-2\mathrm{\varphi x}\right)\right\}}^{0.5}\left[2\varphi^{2}S^{2}\exp\left(-2\mathrm{\varphi x}\right)-1\right]}{{\left\{1+\varphi^{2}S^{2}\exp\left(-2\mathrm{\varphi x}\right)\right\}}^{3}},$$

or

$$2\varphi^{2}S^{2}\exp\left(-2\mathrm{\varphi x}\right)-1=0,$$

resulting in

$$\exp\left(-2\mathrm{\varphi x}\right)=\frac{1}{2\varphi^{2}S^{2}},$$

from which we find

$$-2\mathrm{\varphi x}=\ln\left(\frac{1}{2\varphi^{2}S^{2}}\right),$$

or

(2.0.12) $$x=\frac{\ln\left(\mathrm{\varphi S}\sqrt{2}\right)}{\varphi }$$

Substitution in equation (2.0.1) gives:

(2.0.13) $$y=\frac{1}{\varphi \sqrt{2}}$$

(2.0.14) $$\frac{\mathrm{dy}}{\mathrm{dx}}=-\frac{1}{\sqrt{2}}$$

Notably, the euthyroid set point has been derived from the model parameters of the HP function with coordinates on the FT4-TSH plane given by the ‘Leow-Goede equations’ as follows:

(2.0.15) $$\left[\mathrm{FT}4\right]=\frac{\ln\left(\mathrm{\varphi S}\sqrt{2}\right)}{\varphi }$$

(2.0.16) $$\left[\mathrm{TSH}\right]=\frac{1}{\varphi \sqrt{2}}$$

### Physiological theory of the homeostasis process

**Figure 1:**
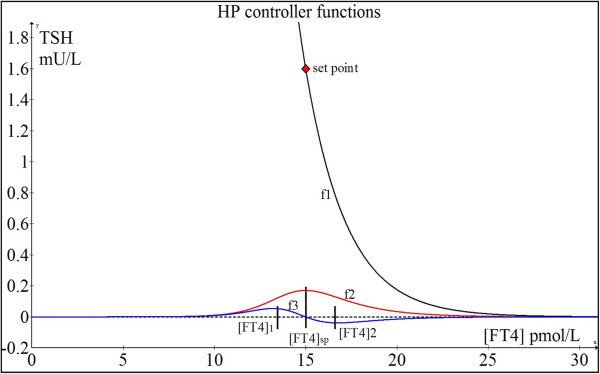
**HP function (f1), curvature function (f2) and derivative of f2 (f3).** For illustrative purposes, [FT4]sp (set point) and [TSH]sp (set point) which respectively represent the values of [FT4] and [TSH] where the euthyroid set point occurs are 15 pmol/L and 1.6 mIU/L respectively. These set point values correspond to $\varphi =\frac{1}{\left[\mathrm{TSH}\right]\sqrt{2}}=0.442$ and *S* = [*TSH*]exp(*φ*[*FT*4]) = 1211.

In Figure 1, the functions of the HP (f1) black curve, the function for the curvature of f1: (f2) red curve and the derivative of the curvature (f3) blue curve are shown in one graph. In this example, the reference ranges used are 10 ≤ [FT4] ≤ 20 pmol/L and 0.4 ≤ [TSH] ≤ 4 mU/L.

If we write for the variable [*FT*4] = *x*

The curvature function:

(2.0.17) f2=K=φ2Sexp−φx1+φ2S2exp−2φx32

The theory for the maintenance of homeostasis around the set point is as follows.

We investigate the second derivative of K:

(2.0.18) $$\frac{d^{2}K}{dx^{2}}=0$$

Here, we find two extremes of f3: at [FT4]1 and [FT4]2 which occurs respectively below and above [FT4]sp, as indicated in Figure 1. [FT4]1 and [FT4]2 function as repulsion boundaries signaling the HP unit to fine-tune the feedback regulation of TSH secretion. When the [FT4] level is increasing and passes [FT4]sp, the HP unit measures both [FT4] and the gradient of f3, and uses the criteria of [FT4]2 together with the change in gradient of f3 from negative through zero to positive to disable the thyroid hormone production when the [FT4] concentration rises beyond [FT4]2. The thyroid hormone production rate will thus decelerate and the [FT4] concentration will accordingly decrease. Then the decline of [FT4] will cross [FT4]sp and fall towards [FT4]1. The HP unit then detects the [FT4]1 level and a change in gradient of f3 from negative through zero to positive to enable an up-regulation of [TSH] to counteract this situation, thereby resulting in an increase in [FT4] again towards [FT4]sp. This dynamic control mechanism based on gradient-curvature detection is quite similar to a modulating regulator with hysteresis as found in advanced thermostats. Essentially, the point of maximum curvature of the HP curve provides the highest sensitivity for [TSH] responses to changes in [FT4] levels such that the vector of [FT4] is always dynamically poised towards a predetermined stable euthyroid homeostatic set point deemed optimal for survival.

### Application of control theory to the optimal euthyroid set point condition

We can better appreciate and understand this robust control and calibration of [FT4] and [TSH] by an intact HPT axis towards euthyroidism in a healthy biological system by applying the notion of a control system represented by the HP unit and the thyroid coupled in a feedback loop. The control system is governed by the HP controller characteristic, unique for every individual like a fingerprint. In this control loop, the HP is the main ‘master’ controller and the thyroid behaves as a ‘slave’ accommodating to the secretory demands, both in rate and magnitude, resulting in two independent thyroid secretory parameters, A and α, such that the loop gain is optimized for the set point position. The loop gain is defined as the modulus of the product of the HP gain factor and the thyroid gain factor. The value of the loop gain is dimensionless and has to be greater than unity in order to maintain optimal and stable control [25]. The loop gain has an optimum coinciding with the set point for stability of the feedback loop which will be proved.

The HP transfer function as mentioned in section *Parameterization of the negative exponential HP characteristic*:

(2.0.19) $$\left[\mathrm{TSH}\right]=S\exp\left(-\varphi \left[\mathrm{FT}4\right]\right)$$

The HP gain factor is defined as the first derivative of the HP transfer characteristic noted as

(2.0.20) $$G_{\mathrm{HP}}=\frac{d\left[\mathrm{TSH}\right]}{d\left[\mathrm{FT}4\right]}=-\mathrm{\varphi S}\exp\left(-\varphi \left[\mathrm{FT}4\right]\right)=-\varphi \left[\mathrm{TSH}\right]$$

The thyroid transfer characteristic is derived from a Michaelis-Menten model [26]. This model can be further modified to a saturating exponential expression which provides a more realistic representation of the thyroid behavior and can be parameterized using two independent model parameters, *A* and *α*. Parameter *A* defines the upper asymptotic secretory [FT4] level and *α* represents the steepness with which the saturation level is reached as a function of the variable [TSH]. The output signal of the thyroid is represented by [FT4]. The function is written as:

(2.0.21) $$\left[\mathrm{FT}4\right]=A\left\{1-\exp\left(-\alpha \left[\mathrm{TSH}\right]\right)\right\}$$

Here, A and α are the independent model parameters of the thyroid transfer function.

The thyroid gain factor *G*_*T*_ can be determined as:

(2.0.22) $$G_{T}=\frac{d\left[\mathrm{FT}4\right]}{d\left[\mathrm{TSH}\right]}=-\mathrm{\alpha A}\exp\left(-\alpha \left[\mathrm{TSH}\right]\right)$$

The loop gain *G*_*L*_ is defined as the modulus of the product |*G*_*HP*_*G*_*T*_| resulting in:

(2.0.23) $$G_{L}=\left|G_{\mathrm{HP}}G_{T}\right|=\varphi \left[\mathrm{TSH}\right]\mathrm{\alpha A}\exp\left(-\alpha \left[\mathrm{TSH}\right]\right)$$

We note that *G*_*L*_ is solely a function of the variable [TSH]. We can examine the properties of *G*_*L*_ related to an optimum value. For this purpose, we determine the first derivative of *G*_*L*_ to [TSH] and examine the solution when this equals zero.

(2.0.24) $$\frac{dG_{L}}{d\left[\mathrm{TSH}\right]}=\frac{\mathrm{\varphi \alpha A}-\varphi \alpha^{2}A\left[\mathrm{TSH}\right]}{\exp\left(\alpha \left[\mathrm{TSH}\right]\right)}=0$$

The use of [TSH] as the common variable for differentiation should be maintained because the loop gain *G*_*L*_ is a function of that variable. It follows that at optimum *G*_*L*_,

(2.0.25) $$\left[\mathrm{TSH}\right]=\frac{1}{\alpha }$$

Therefore,

(2.0.26) $$\alpha =\frac{1}{{\left[\mathrm{TSH}\right]}_{\mathrm{setpo}\mathrm{int}}}$$

Using the original expression for the thyroid,

(2.0.27) $${\left[\mathrm{FT}4\right]}_{\mathrm{setpo}\mathrm{int}}=A\left\{1-\exp\left(-\alpha {\left[\mathrm{TSH}\right]}_{\mathrm{setpo}\mathrm{int}}\right)\right\},$$

and substituting the result for α, we have:

(2.0.28) $${\left[\mathrm{FT}4\right]}_{\mathrm{setpo}\mathrm{int}}=A\left\{1-\exp\left(-\frac{{\left[\mathrm{TSH}\right]}_{\mathrm{setpo}\mathrm{int}}}{{\left[\mathrm{TSH}\right]}_{\mathrm{setpo}\mathrm{int}}}\right)\right\}=A\left\{1-\frac{1}{e}\right\}=0.632A,$$

with e = 2.7182, we then find A:

(2.0.29) $$A=\frac{{\left[\mathrm{FT}4\right]}_{\mathrm{setpo}\mathrm{int}}}{0.632}$$

(2.0.30) $$G_{L}=\frac{\mathrm{\alpha A\varphi}\left[\mathrm{TSH}\right]}{\exp\left(\alpha \left[\mathrm{TSH}\right]\right)}$$

(2.0.31) $${G_{L}}_{\text{max}}=\frac{\mathrm{\varphi A}}{e}$$

Based on these relationships, the thyroid will operate such that the loop gain will be optimal at the set point. This means the thyroid accommodates to both the demands of secretory amplitude (A) and steepness (α). In a calculation example, we demonstrate the properties of the optimum in the loop gain belonging to the set point, [FT4] = 15 pmol/L and [TSH] = 1.6 mU/L.

This results in $\alpha =\frac{1}{\left[\mathrm{TSH}\right]}=\frac{1}{1.6}=0.625$ and from (2.0.29), we derive:

$$A=\frac{{\left[\mathrm{FT}4\right]}_{\mathrm{setpo}\mathrm{int}}}{0.632}=\frac{15}{0.632}=23.73$$

As earlier shown in Figure 1, we found according to the set point equations S = 1211 and *φ* = 0.442.

The exponential thyroid model is thus:

$$\left[\mathrm{FT}4\right]=23.73\left\{1-\exp\left(-0.625\left[\mathrm{TSH}\right]\right)\right\}$$

The thyroid function to be depicted in an inverted axis superimposed into the same plot as the HP function, because the common variable is [TSH], results in:

$$\left[\mathrm{TSH}\right]=1.6\ln\left(\frac{15}{15-0.632\left[\mathrm{FT}4\right]}\right)$$

The loop gain function was derived before as:

(2.0.32) $$G_{L}=\left|G_{\mathrm{HP}}G_{T}\right|=\varphi \left[\mathrm{TSH}\right]\mathrm{\alpha A}\exp\left(-\alpha \left[\mathrm{TSH}\right]\right)$$

**Figure 2:**
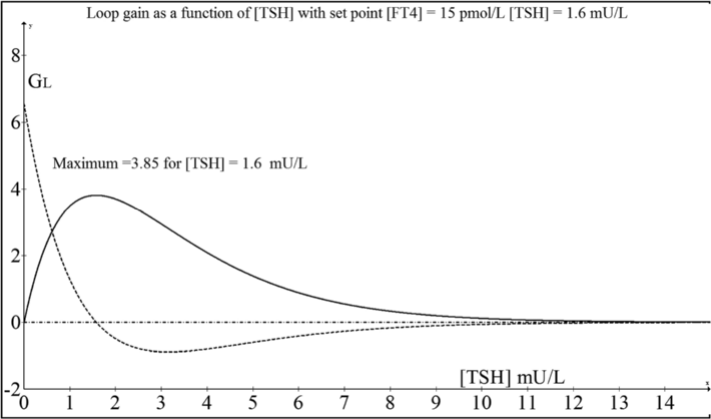
**Plot of loop gain as a function of TSH (solid line).** This shows a maximum loop gain at the set point while the first derivative of the loop gain is illustrated by the dotted line.

As depicted in Figure 2, the first derivative of *G*_*L*_ declines to zero exactly over the [TSH] value of 1.6 mU/L at which the maximum of the loop gain occurs. As can be checked by the application of:

(2.0.33) $${G_{L}}_{\text{max}}=\frac{\mathrm{\varphi A}}{e},$$

this maximum loop gain equals 3.85.

This shows that the absolute value of the loop gain decreases when [TSH] deviates from the set point value. This negative feedback loop itself will maintain this stable condition for homeostasis.

The euthyroid set point in this example is shown by the negative exponential curve defined by parameters S = 1211 and φ = 0.442 (ie. TSH = 1211 exp (−0.442[FT4])). From these parameters, the set point at the maximum curvature of this HP curve is: [FT4] = 15 pmol/L and [TSH] = 1.6 mU/L. When we substitute [FT4] = 15 pmol/L into equation (2.0.21), we find [TSH] = 1.6 mU/L, which also satisfies the condition of the set point occurring at the intersection of the HP characteristic curve and the inverted thyroid characteristic derived from (2.0.21):

(2.0.34) $$\left[\mathrm{TSH}\right]=-\frac{1}{\alpha }\ln\left(\frac{A-\left[\mathrm{FT}4\right]}{A}\right)$$

**Figure 3:**
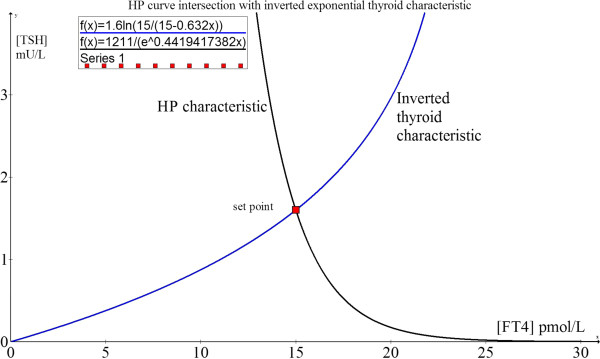
**The homeostatic euthyroid equilibrium point.** The set point occurs at the intersection of the HP (black) and the inverted thyroid (blue) functions coinciding with the maximum curvature of the HP function.

This intersection (Figure 3) is the mathematical solution of these two simultaneous non-linear equations and corresponds to the concentration of [TSH] which stimulates just the appropriate [FT4] output which in turn feedback negatively to keep [TSH] exactly at this stationary input level. Hence, this is a necessary and sufficient condition for stable equilibrium and physiologically represents the homeostatic euthyroid set point, thereby proving yet again that this state indeed overlies the point of maximum curvature of the HP function. The set point from optimal control perspectives is analogous thermodynamically to the position of lowest potential energy for maximum system stability and efficiency in the steady state as also demonstrable in other physiological systems [27].

### Sensitivity analysis of exponential model parameters

Based on the formulation describing the [FT4]-[TSH] characteristic, when two (or more) measured points (eg. ([FT4]1, [TSH]1) and ([FT4]2, [TSH]2)) from an individual are distinctly separated on the [FT4]-[TSH] plane such as typically observed during the dose titration process of treatment, it is possible to calculate the HP characteristic based on parameters S and φ as follows:

(2.0.35) $$\varphi =\left(\frac{1}{\left({\left[\mathrm{FT}4\right]}_{1}-{\left[\mathrm{FT}4\right]}_{2}\right)}\right)\ln\left(\frac{{\left[\mathrm{TSH}\right]}_{2}}{{\left[\mathrm{TSH}\right]}_{1}}\right)$$

(2.0.36) $$S={\left[\mathrm{TSH}\right]}_{1}\exp\left(\varphi {\left[\mathrm{FT}4\right]}_{1}\right)={\left[\mathrm{TSH}\right]}_{2}\exp\left(\varphi {\left[\mathrm{FT}4\right]}_{2}\right)$$

**Figure 4:**
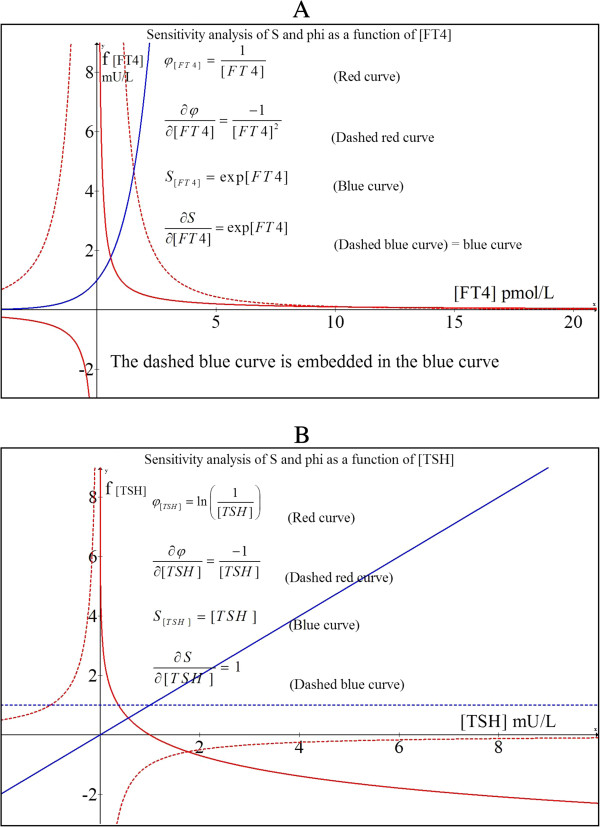
Sensitivity analysis of S and φ A) Parameter variations as a function of [FT4] and B) Parameter variations as a function of [TSH].

We will distinguish the various dependencies of φ and S in relation to [FT4] and [TSH] as follows. The dependency of φ for variations in [FT4] holding [TSH] constant, can be written as:

(2.0.37) $$\varphi =\frac{1}{\left[\mathrm{FT}4\right]}$$

in Figure 4A indicated as red curve.

The dependency of φ for variations in [TSH] holding [FT4] constant, can be written as

(2.0.38) $$\varphi =\ln\left(\frac{1}{\left[\mathrm{TSH}\right]}\right)$$

This dependency is depicted in Figure 4B as a solid red curve.Hence, the variations in φ as a function of [FT4] fluctuations (ie. equation (2.4.1)) can be depicted by the solid red graph shown in Figure 4A while the effect of fluctuations of [TSH] on φ (ie. equation (2.4.2)) is shown by the solid red graph of Figure 4B.

From these graphs, we can determine the partial derivatives which represent the gain factor for small deviations around a certain point on either the blue or the red curve. Thus,

(2.0.39) $$\frac{\partial \varphi }{\partial \left[\mathrm{FT}4\right]}=\frac{-1}{{\left[\mathrm{FT}4\right]}^{2}},$$

shown in Figure 4A

(2.0.40) $$\frac{\partial \varphi }{\partial \left[\mathrm{TSH}\right]}=\frac{-1}{\left[\mathrm{TSH}\right]},$$

shown in Figure 4B.

It can be readily appreciable that the degree of error in the estimation of φ escalates rapidly when FT4 < 2 pmol/L, whereas variations in [TSH] < 0.5 mU/L will contribute to relatively large errors in φ. For S, we find the following:

(2.0.41) $$\frac{\partial S}{\partial \left[\mathrm{TSH}\right]}=1$$

as a straight dashed blue line in Figure 4B and

(2.0.42) $$\frac{\partial S}{\partial \left[\mathrm{FT}4\right]}=\exp\left[\mathrm{FT}4\right],$$

as depicted in Figure 4A in dashed blue which fully coincides with the blue curve.

Errors in S estimation are independent of [TSH] but escalate with increasing [FT4]. Fortunately, modern FT4 assays are robust to errors while third generation TSH assays are sensitive and precise.

Clinical examples in this paper illustrate that S and φ estimates are reassuringly accurate in practice for the purpose of elucidating the set point to guide individualized therapeutic targets.

### Ethical approval, laboratory assays and statistical analysis

For the purpose of clinical validation of this theoretical framework, a group of normal euthyroid individuals (N = 14) were randomly sampled and their TFTs were traced to assess if [FT4] and [TSH] of each individual essentially remained stable over time (Table 1). Another patient group (N = 70) with thyroid disease on medication were randomly selected to validate the set point theory. All clinical dataset in this study was acquired with ethical approval (National Healthcare Group Domain Specific Review Board (DSRB ‘C’) ethics committee, C/2011/02012) from individuals under follow-up by the co-author (MKSL) in the Department of Endocrinology at Tan Tock Seng Hospital of Singapore. Serum TSH and FT4 were assayed in the clinical chemistry laboratory of the same hospital using manufacturer-supplied standard reagents, calibrators and controls from Beckman Coulter, Krefeld, NRW, Germany, on two Beckman Coulter DxI800 automated immunoassay analyzers. The intra-assay and inter-assay CVs for both TSH and FT4 are less than 10% and the normal reference intervals for FT4 and TSH are 8–21 pmol/L and 0.34-5.6 mIU/L respectively.

**Table 1:** Healthy euthyroid subjects showing their stability of their TFTs over time

**Patient**	**TFT paired results (FT4, TSH) over time**						**Similarity metric**
1 (S25)	Date:	Apr ‘07	Aug ‘09	Dec ‘09	Jan ‘10	Mar ‘11	0.3679
	FT4:	9	9	8	10	10	
	TSH:	1.59	1.71	1.34	1.87	1.69	
2 (S26)	Date:	Sep ‘01	May ‘02				0.9861
	FT4:	12	12				
	TSH:	0.89	0.71				
3 (S27)	Date:	Jun ‘07	Apr ‘ 08	Feb ‘10			0.5504
	FT4:	11	11	12			
	TSH:	1.00	1.03	1.02			
4 (S42)	Date:	Nov ‘10	Jun ‘12	Feb ‘13			0.6138
	FT4:	10	10	10			
	TSH:	3.25	2.40	2.43			
5 (S18)	Date:	Apr ‘06	Jul ‘12				0.5858
	FT4:	10	11				
	TSH:	1.89	1.90				
6 (S20)	Date:	May ‘97	Sep ‘00	Jul ‘05	Feb ‘08	Oct ‘11	0.4114
	FT4:	16	15	16	16	15	
	TSH:	1.31	1.08	1.57	1.24	1.35	
7 (S22)	Date:	Aug ‘02	Oct ‘11	Jun ‘12			0.5484
	FT4:	13	14	12			
	TSH:	1.16	1.46	1.26			
8 (S23)	Date:	Feb ‘10	Nov ‘10	Nov ‘11			0.3003
	FT4:	12	11	11			
	TSH:	0.71	0.85	0.83			
9 (S10)	Date:	Apr ‘04	Dec ‘05	Nov ‘06	Aug ‘07		0.4109
	FT4:	13	11	11	10		
	TSH:	0.92	1.87	1.73	1.96		
10 (S13)	Date:	Aug ‘03	Mar ‘06	Jul ‘12			0.3729
	FT4:	10	9	11			
	TSH:	1.66	1.38	1.68			
11 (S15)	Date:	Oct ‘06	Jan ‘09	Dec ‘09	Oct ‘10	Sep ‘12	0.4074
	FT4:	13	11	11	12	12	
	TSH:	0.84	0.69	0.90	0.77	0.87	
12 (S09)	Date:	Nov ‘05	Oct ‘06	Jul ‘11	Jun ‘12		0.4647
	FT4:	11	10	9	10		
	TSH:	1.53	1.99	1.68	1.85		
13 (S49)	Date:	Mar ’11	Mar ‘13				0.5857
	FT4:	12	13				
	TSH:	0.84	0.8				
14 (S53)	Date:	Sep ’05	Mar ’12	Jul ‘13			0.3492
	FT4:	13	11	13			
	TSH:	1.18	2.45	1.83			

Normal [FT4] reference range: 8 ≤ [FT4] ≤ 21 pmol/L and for [TSH]: 0.34 ≤ [TSH] ≤ 5.6 mU/L.

We have to take into account that the datasets in the results section are not optimized for accuracy and reproducibility as we have discussed in a recent paper about the optimal conditions for the HP model [22]. Nonetheless, the data serve well to prove our set point conjecture. All statistical analyses were conducted using the statistical program Stata (Release 13, StataCorp. College Station; Texas: StataCorp LP).

## Results

### Stability and uniqueness of the HPT axis euthyroid set point

Before we proceed to apply the theory of the homeostatic euthyroid set point as described above to patients with thyroid hormonal disorders, it is insightful to understand the properties of the euthyroid set point as it functions in healthy individuals. Assuming that this set point has been programmed by factors including growth, body weight, energy supply, metabolic rate and the complex nexus of genes-environment interactions such that its operating parameters guarantee a state of optimal health, it stands to reason that the [FT4] and [TSH] surrounding the set point should remain relatively stable even over an extended period of time across the lifespan of the individual. This euthyroid set point (or ‘set range’) is thus expected to be tightly regulated with a physiological window far narrower than the usual normal population reference ranges of [FT4] and [TSH] and is likely to be unique in any given person.

**Figure 5:**
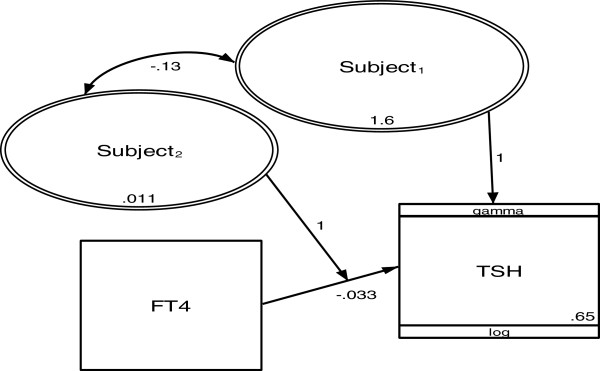
**Multi-level regression model of the dataset in Table**1**(gamma distribution and log link; random intercept and slope model).** TFTs are level 1 while subjects are level 2. Rectangles represent observed variables and ovals represent latent variables. Numbers represent variance, covariance, regression coefficient and constant.

It is remarkable that each person, when healthy, continues to show TFTs that are closely clustered together even after a decade later as shown in Table 1. To evaluate closeness of 2-D data points, the Euclidean distance (ED) is employed [28]. This can be normalized to a ‘similarity metric’, a basic measurement of the degree to which two objects are alike, by taking the reciprocal of ED:

(2.0.43) $$\mathrm{Similarity}\;\mathrm{metric}\;=\;\frac{1}{1+\mathrm{ED}},$$

such that the values returned by this computation lie between 0 and 1, whereby 0 means no similarity and 1 implies complete similarity [29]. Virtually every individual exceeded 0.3, indicating a very strong intra-personal TFT clustering effect. Furthermore, statistical analysis using the multi-level regression with a general structural equation modeling framework (GLLM) [30] revealed the heterogeneity between subjects to be 0.0107 (95% CI: 0.0029-0.03975, p < 0.05). Each TFT pair is both tightly clustered intra-individually and distinctly separate from one another (Figure 5), supporting our hypothesis that everyone has a unique and stable euthyroid set point.

Given that the [FT4] data in Table 1 are either rounded off or had decimals truncated results from original assay readouts (often up to one decimal accuracy), these values can be interpreted as [*FT*4] ± 1 [6,22]. For [TSH] in Table 1, we can assume that the readings are well within the normally expected diurnal range of [TSH] variations.

### Clinical validation and application of the set point theory

As shown above, there is strong evidence that the euthyroid set point is relatively constant and stable in most healthy people at least over a span of one to two decades. From a teleological perspective, it benefits long-term health for physiological functions driven by metabolically potent thyroid hormones to be optimally regulated within feedback threshold hormonal levels encompassing the euthyroid set point and its surrounding narrow neighborhood for maximal disease-free survival. Our theoretical modeling has clearly demonstrated the natural tendency of such a set point to be robustly defended at the point of maximum curvature of the HP function. Clearly, it is still crucial to validate the model with supporting clinical data. In an intact HPT axis, the closed feedback loop controls the [FT4] and [TSH] to the set point and thus obscures the unique negative exponential HP function necessary to test the theory. It requires a pathological thyroid state to break open the closed loop and unravel the HP characteristic for testing. A number of independent methods as listed below may be employed by researchers keen to study the validity of this set point theory, with the caveat that some could be considered challenging to perform, logistically unfeasible or possibly ethically untenable depending on the circumstances.

### Methods of clinical validation

In ascending order of difficulty and feasibility, the following are various methods of clinical validation strategies that may be employed to test the set point theory:

(1) Tracking the TFTs during the treatment of those with total thyroidectomy (eg. thyroid cancer, massive multinodular goiter, etc.) who require life-long L-T4; their original euthyroid pre-operative FT4-TSH levels would be considered their original set points that can be compared to the set points calculated using their plotted HP curves.

(2) Patients with Graves’ disease who successfully achieved a sustained long term clinical remission after successfully weaning off antithyroid drugs; their TFTs during remission may be considered their euthyroid set points which can be compared with the set point values computed from their HP curves while they were previously still on treatment.

(3) Patients presently on anti-thyroid drugs for hyperthyroidism or L-T4 for hypothyroidism with a prior historical record of previously normal euthyroid FT4-TSH data long before the onset of thyroid disease that subsequently requires definitive thyroid-specific treatment and dose adjustments, whereupon their computed set points on their HP curves can be matched against their original normal FT4-TSH data.

(4) Searching existing epidemiologic literature with published laboratory data documenting the natural history of those screened positive for thyroid autoimmune markers associated with an euthyroid state initially and who then subsequently develop thyroid disease requiring treatment.

(5) Patients who received radiation therapy of the neck for cancers (eg. lymphomas, parotid gland carcinoma or nasophayngeal cancer) who then developed hypothyroidism allowing HP curves to be plotted and set points computed; any euthyroid TFTs pre-radiotherapy would serve as a benchmark for comparison with the computed set points.

(6) Euthyroid healthy human volunteers transiently rendered hypothyroid using thionamides and/or administered either L-thyroxine (L-T4) or liothyronine (L-T3) to induce experimental thyrotoxicosis according to ethically approved safe research protocols (depending on the jurisdiction of institutional review boards) in order to generate unique HP curves for set points determination which can then be compared against their own natural set points.

(7) Unusual patients with “euthyroid Graves’ disease”, whose TFTs are consistently within the normal ranges could arguably be considered euthyroid set points due to an equimolar mixture of TSH receptor stimulating and blocking autoantibodies with a ‘tug-of-war’ stoichiometric ratio resulting in euthyroidism; subsequent development of thyrotoxicosis or hypothyroidism due to antibodies imbalance allows HP curves to be plotted for set point computations.

(8) Prospective follow-up of euthyroid people positive for thyroid autoantibodies (with previous euthyroid TFTs prior to developing thyroid autoimmunity) over years until their thyroid dysfunction manifests, after which the calculated set points on their respective HP curves can be compared with their initial euthyroid TFTs.

### Examples of clinical data validating set point calculations

The following clinical data formally illustrate and constitute an elegant proof-of-concept of our set point theory. We demonstrate five case examples (out of a total of N = 70 cases) to allow an in-depth analysis of the set point confirmation with real clinical data. However, the remaining cases are summarized graphically as shown in Figure 14A-D and Figure 15.

#### Case 1: 53 yr old Chinese woman P11 (LMC)

This patient has bipolar affective disorder treated with lithium by her psychiatrist two years ago. This precipitated overt symptomatic hyperthyroidism. Serial paired FT4 (in pmol/L) and TSH (in mU/L) and drug dosing (Rx) with carbimazole (CMZ in mg) and levothyroxine (L-T4 in μg) are:

Her thyroid was ablated with 18 mCi of radioiodine-131, following which CMZ could be stopped while LT4 started. She has some cold intolerance when FT4 was 10 pmol/L but felt better as her FT4 started rising above 10 pmol/L.

**Figure 6:**
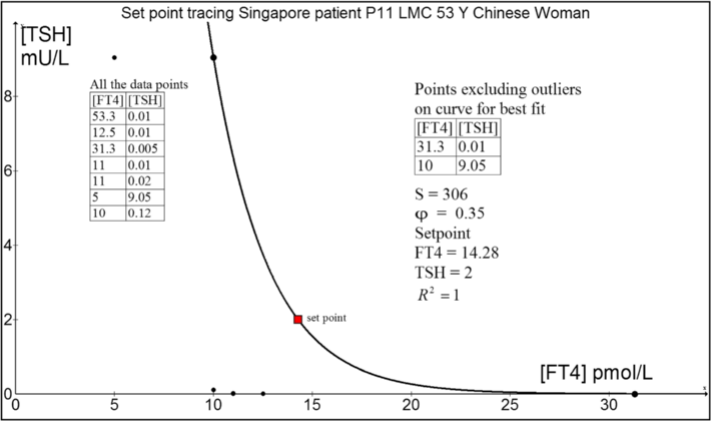
Set point tracing of patient P11 (LMC).

Her pre-lithium euthyroid TFT six years ago was: FT4 = 14.6 pmol/L and TSH = 1.72 mU/L which matched the calculated set point closely (FT4 = 14.28 pmol/L and TSH = 2.00 mU/L) (Figure 6). The outliers falling outside the expected range of the model selection were excluded [24].

#### Case 2: 56 yr old Chinese woman P53 (AL)

**Figure 7:**
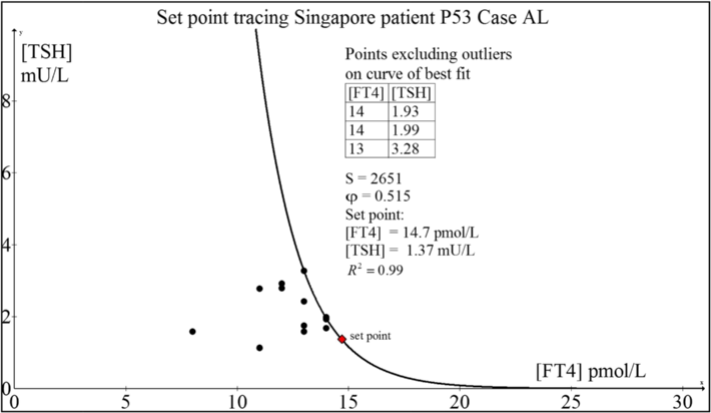
Set point tracing of patient P53 (AL).

This patient was diagnosed with Graves' disease two months after the development of pretibial myxoedema. After a two-year course of antithyroid drug (ATD) therapy with CMZ, her serum TSH receptor autoantibody (TRAb) fell below immunoassay detection limit of < 0.4 U/L when CMZ was tapered off and she remained in full remission till today.The computed euthyroid set point is FT4 = 14.7 pmol/L and TSH = 1.37 mU/L (Figure 7). This patient’s own historical TFT record showed a euthyroid FT4 = 16.1 pmol/L and TSH = 1.43 mU/L done 15 months before she contracted Graves’ disease. There is relatively satisfactory agreement between the predicted set point and the actual euthyroid set point of the patient. The excluded outliers are out of the range of the model selection because of significant hysteresis effect whereby TSH gene expression remains subnormal for any given FT4 following recent hyperthyroidism.

#### Case 3: 69 yr old Chinese woman P31 (SSPG)

**Figure 8:**
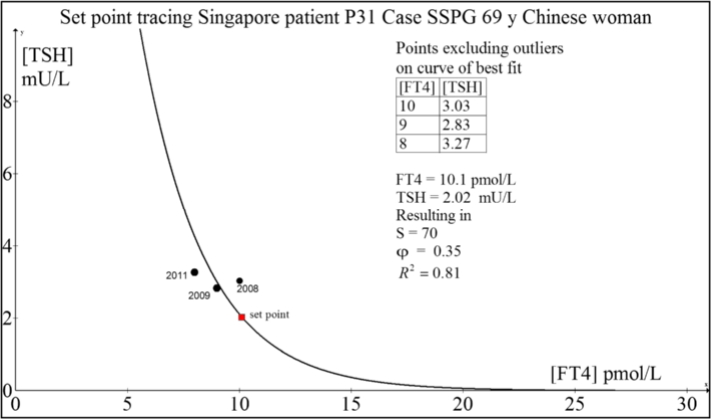
Set point tracing of patient P31 (SSPG).

This woman was well till some 5 years ago when she consulted a rheumatologist for stenosing tenosynovitis and carpal tunnel syndrome. As part of the investigative workup, TFT was done which showed borderline FT4 and elevated anti-thyroperoxidase autoantibodies (TPO Ab > 500 U/L). Given that TPO Ab predicts a risk of thyroid failure, annual TFTs were serially monitored. Her TFT a decade ago during a health screening was normal: FT4 = 12 pmol/L and TSH = 1.53 mU/L.The computed set point is FT4 = 10.1 pmol/L and TSH = 2.02 mU/L (Figure 8). This estimate is roughly concordant with her original euthyroid set point.

#### Case 4: 76 yr old Chinese man P68 (KST)

**Figure 9:**
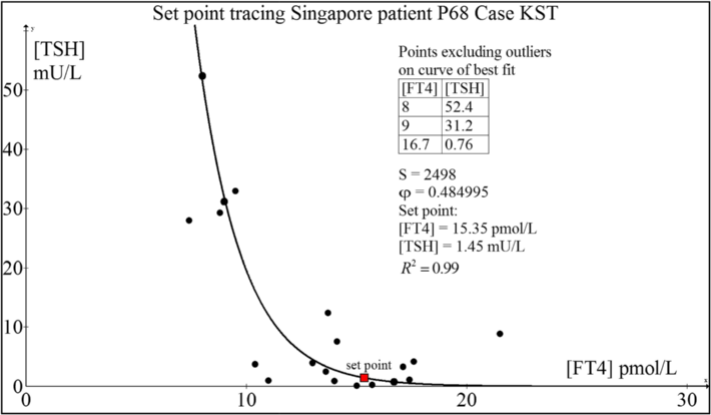
Set point tracing of patient P68 (KST).

This patient has a history of laryngeal squamous cell carcinoma for which he underwent total laryngectomy with radical neck dissection followed by adjuvant chemo- and radiotherapy. His pre-surgery, pre-radiotherapy TFT was FT4 = 17.8 pmol/L and TSH = 0.88 mU/L which can be considered his original euthyroid set point. His subsequent TFTs post-cancer treatment were:From the computed set point (FT4 = 15.35 pmol/L and TSH = 1.45 mU/L) (Figure 9) and his initial euthyroid TFT (FT4 = 17.8 pmol/L and TSH = 0.88 mU/L), it can be concluded that the predicted set point was reasonably close to his original normal TFT.

#### Case 5: 52 yr old Chinese woman P45 (LBH)

This woman was healthy and well until 2004 when she discovered a painless right thyroid swelling.

**Figure 10:**
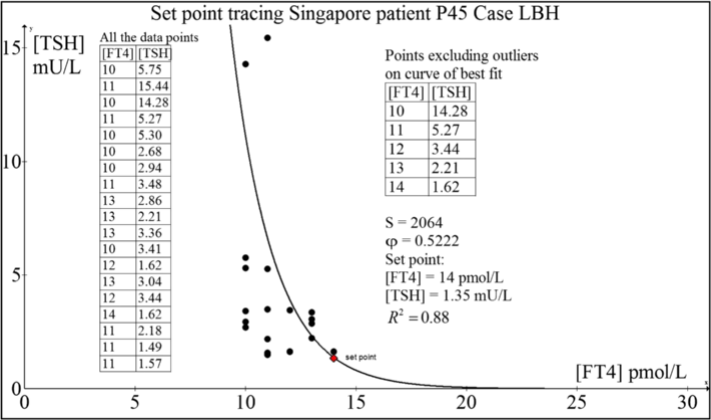
Set point tracing of patient P45 (LBH).

She was clinically and biochemically euthyroid (pre-operative FT4 = 13.0 pmol/L and TSH = 1.35 mU/L). Thyroid ultrasonography revealed a solid-cystic nodule and fine needle biopsy showed a suspicious follicular lesion. She underwent right thyroid lobectomy which showed nodular goitre with cystic degeneration but no malignancy. Subsequent regular monitoring with serial TFT showed development of subclinical hypothyroidism which progressed over a span of two years to overt hypothyroidism requiring LT4 replacement as she fatigued readily and put on weight easily.Her computed set point was FT4 = 14.0 pmol/L and TSH = 1.35 mU/L which matched her original TFT very closely (Figure 10).

### Case examples when original normal euthyroid set point is unavailable

In the common clinical setting when patients with either hyperthyroidism or hypothyroidism present to the medical doctor, there is no prior TFT done by the patient previously before the disease onset. As such, the set point theory can be put to the acid test to evaluate if the patients’ health outcomes are optimized or improved when their thyroid medications are dose titrated till the their TFTs approach the computed set points.

#### Case 1: 38 yr old Chinese man P19 (NWC)

**Figure 11:**
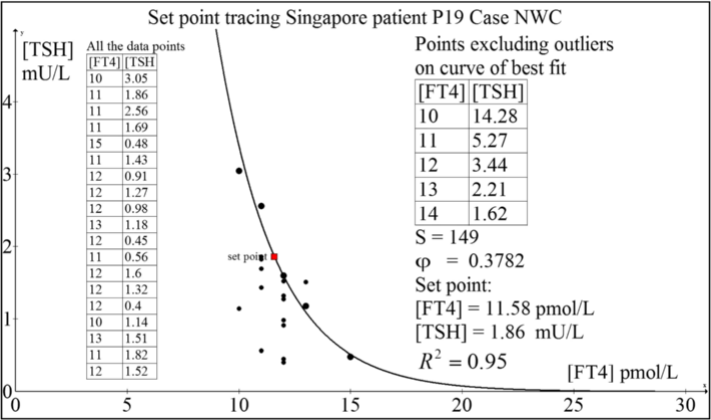
Set point tracing of patient P19 (NWC).

This man was diagnosed with Graves’ disease in February 2002 when he presented with thyrotoxic periodic paralysis associated with severe hypokalemia (plasma potassium = 1.8 mmol/L) due to transcellular potassium shift precipitated by hyperthyroxinemia.His initial TSH receptor antibody (TRAb) level was 9.7 U/L. After 46 months of daily propylthiouracil (PTU) therapy, he was weaned off PTU completely when TRAb became undetectable and has remained euthyroid ever since December 2005. Regular follow-up revealed stable TFTs with FT4 ~ 11–13 pmol/L and TSH ~ 1.3-1.9 mIU/L approximately. These values may be reflective of his original HPT axis euthyroid set point. Using our minimal model, his set point was computed as: FT4 = 11.58 pmol/L and TSH = 1.86 mIU/L (Figure 11). This predicted set point agrees closely with our clinically observed euthyroid estimates. Indeed, he felt positively nursed back to health state at this present time.

Analyzing the loop gain at this predicted set point using control theory and applying equation (2.3.4), we have:

$$\begin{array}{ll} G_{L} & =\left|G_{\mathrm{HP}}G_{T}\right|=\varphi \left[\mathrm{TSH}\right]\mathrm{\alpha A}\exp\left(-\alpha \left[\mathrm{TSH}\right]\right)\\ & =\left(0.3782\right).\left(1.86\right).\left(0.538\right).\left(18.32\right).\exp\left[\left(-0.538\right).\left(1.86\right)\right]\\ & =2.55 \end{array}$$

Since this loop gain exceeds unity, the negative feedback loop is operational and the set point is stable in this patient’s HP control system.

#### Case 2: 77 yr old Chinese woman P22 (LBG)

**Figure 12:**
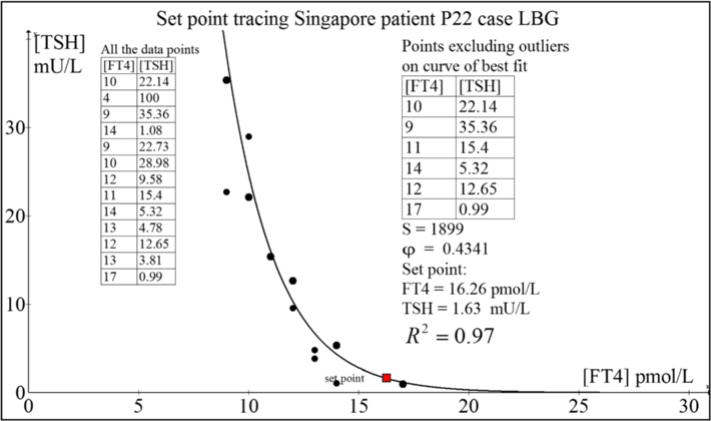
Set point tracing of patient P22 (LBG).

This elderly woman presented with classical primary hypothyroidism due to Hashimoto’s thyroiditis, including weight gain, lethargy, cold intolerance, dry skin and constipation. Her serum thyroperoxidase autoantibodies (TPO Ab) exceeded 500 IU/L and anti-thyroglobulin (anti-Tg Ab) titre was greater than 102,400. She was initiated on L-T4 replacement at 25 mcg daily and titrated gradually to 100 mcg daily. Unfortunately, she had memory lapses which led to suboptimal compliance to L-T4 dosing as we subsequently discovered. Hence, her L-T4 dose was eventually fixed at 100 mcg daily till she finally attained clinical and biochemical euthyroidism.She persistently experienced cold intolerance despite TFTs within the normal ranges. Remarkably, she felt normal for the very first time when FT4 = 17 pmol/L and TSH = 0.99 mIU/L was attained through full medication compliance, thus implying this to be her euthyroid set point. This agrees closely with the computed set point: FT4 = 16.26 pmol/L and TSH = 1.63 mU/L (Figure 12). The loop gain at the set point is 4.11, confirming a stable position (ie. homeostatic equilibrium).

#### Case 3: Chinese woman P20 (LPB)

**Figure 13:**
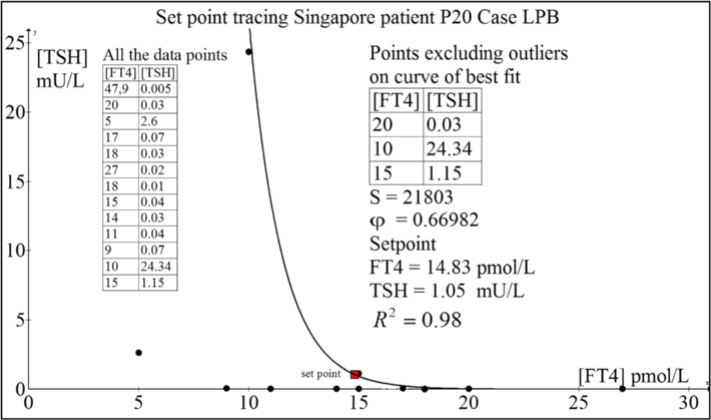
Set point tracing of patient P20 (LPB).

This woman has underlying Graves’ disease on CMZ for 18 months before she underwent total thyroidectomy. Post-operatively, she developed overt hypothyroidism while on L-T4 50 μg daily. L-T4 was thus escalated to 75 μg daily which stabilized her at FT4 = 15 pmol/L and TSH = 1.15 mU/L and rendered her fully asymptomatic. Thus, this probably represented her euthyroid set point though she had no pre-disease TFT to countercheck. Her computed her set point was FT4 = 14.83 pmol/L and TSH = 1.05 mU/L (Figure 13), which was nearly a perfect match with the observed euthyroid TFT.

The calculated loop gain here equals 5.78, indicative of a very stable position of homeostasis.

#### Clinical evidence supporting the exponential model for set point computations

It has been established for decades that the log-linear relationship seems to operate very well in correlating the [TSH]-[FT4] hormone pair in the HPT axis [15-21]. However, it is noteworthy that some very recent papers have surfaced that challenge this notion. The models being proposed as more accurate varied from error function, fourth power polynomials to negative sigmoidal curves with inflexion points, and are all virtually based on aggregate analyses of clinical data of hundreds of thousands of different individuals [31-34]. Interestingly, the latter negative sigmoidal curves model has in fact been predicted by fundamental mathematical modeling [14]. Crucially, this negative sigmoidal profile can be shown to be practically approximated by a parameterizable log-linear model when the ranges of [TSH] and [FT4] are confined to values not extending to pathological extremes associated with the phenomenon of hysteresis [14,22].

**Figure 14:**
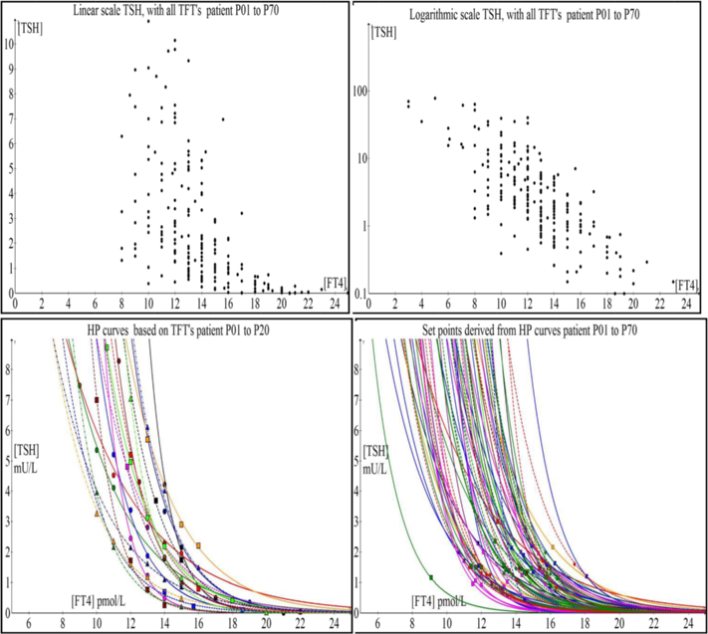
Scatter plots and associated HP curves A) TSH vs. FT4 (N=70) B) log TSH vs. FT4 (N=70) C) HP curves (N=20) and D) HP curves with individual set points (N=70).

**Figure 15:**
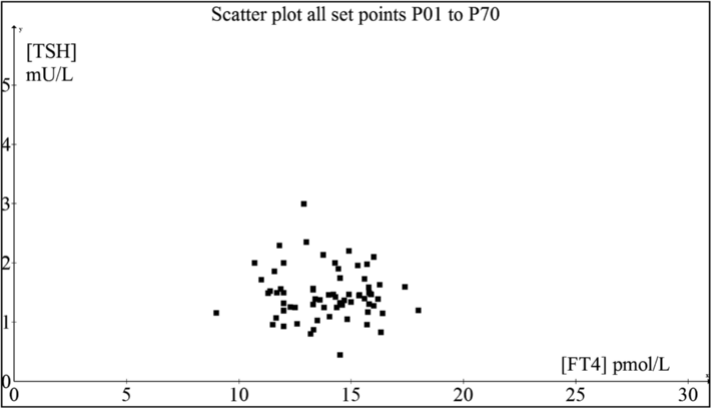
Graph showing the scatter-plot of the computed set points of all cases (N = 70).

The key commonality in terms of weakness in methodology in all these recent papers is the crude attempt to fit different mathematical functions into a fuzzy and unrelated data cloud belonging to different individuals collectively bunched together for analysis at the population level. This leads to the illusion that the relationship of [TSH] and [FT4] is not a log-linear one. As such composite data introduce variations and errors that are typically analyzed statistically using means/medians of these data aggregates, the underlying physiological signal can be totally masked and misinterpreted due to excessive noise superimposed on the signal [35]. As these TFT paired coordinates looked randomly distributed, various “nonlinear models of best fit” can conceivably be regressed through the scatter-plot (Figure 14A and B). If each person is separately analyzed, the signal-to-noise ratio is markedly improved and the negative exponential curves become unmistakably apparent as elegantly proven when we fit the curves on an individual basis (N = 20) (Figure 14C). In fact, in our entire case series (N = 70), all patients’ individual TFT coupled ‘data pairs’ can be readily fitted with a negative exponential curve with a coefficient of determination (R^2^) well exceeding 80% which strongly supports the parameterizable log-linear relationship (Figure 14D). In every case, the set point can be computed accurately using this minimal negative exponential model and it is evident that most patients have euthyroid set points that are distinctly unique and separate from one another (Figure 15). Notably, the appreciable ‘noisy scatter’ of these euthyroid TFT points (N = 70) bears no trace of resemblance to any negative exponential curve. Yet, an individualized HP curve remarkably passes through each of these set points (Figure 14D). It cannot be overemphasized that basic physiological laws and principles have always been traditionally investigated and enunciated precisely by physiologists at the individual cellular, tissue or organismal level as epidemiological and statistical methods are fundamentally flawed approaches to unravel such biological signals and relationships with any degree of accuracy and confidence.

### Corroboration from an animal model

**Figure 16:**
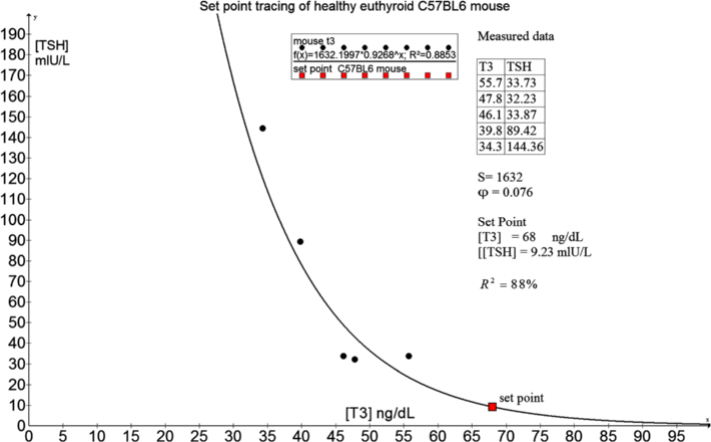
Computation of set point using a C57BL/6 healthy mouse model.

We acknowledge that we were unable to induce thyrotoxicosis and hypothyroidism in healthy human beings with intact HPT axis operating in a closed loop to demonstrate the precision of our set point theory. This clinical experiment itself is not without risks and poses definite bioethical issues which limit our ability to prove the construct the HP curves and apply the set point theory using perfectly healthy human controls. However, one of the author (MKSL) who is a clinician scientist have conducted experiments with SICS (A*STAR) scientists using healthy wild-type adult male C57BL/6 inbred mice in our investigations of perturbing the HPT axis in a murine model. Animal care and experimentation were in accordance with the Guidelines for the Care and Use of Laboratory Animals and were ethically approved by the Institutional Animal Care and Use Committee, Biological Resource Centre (IACUC #120723), Singapore. These mice were housed in husbandry under conventional conditions (23°C, 50%–60% humidity and condition) and maintained with free access to food and water under a 12-h light/dark cycle in compliance to the guidelines of the National Advisory Committee for Laboratory Animal Research (NACLAR) of Singapore. The protocol for induction of hypothyroidism was 0.15% 6-n-propyl-2-thiouracil (PTU) in low iodine chow (TD.95125 from Harlan Laboratories) for 28 days. For induction of thyrotoxicosis, adult mice were treated with intraperitoneal 5 μg of L-T3 (3,3’,5-triiodo-L-thyronine from Sigma-Aldrich, St Louis, MO, USA) for 14 days. As an illustration, we computed the set point and showed that the dataset from a mouse model (serum total T3 in ng/dL and mouse TSH in mU/L) is valid with a goodness-of-fit (R^2^) of 88% (Figure 16). This implies that the maximum curvature theory may be applicable across a wider species of vertebrates and hence may also be used by veterinary doctors and zoologists.

## Discussion

The treatment of patients with hyperthyroidism and hypothyroidism worldwide for many decades till this day has continued to be the enduring clinical practice of titrating the appropriate thyroid medication in order to achieve the therapeutic objective of biochemical euthyroidism as defined by the attainment of [FT4] and [TSH] within the normal population reference limits [36,37]. However, this well established strategy has only recently been subject to scrutiny and critique due to many thyroid patients who persistently complained of residual symptoms despite being rendered ‘euthyroid’ according to the above criteria [4-8], an enigma that challenges the existing notion of what exactly is euthyroidism itself. Distinct time lags between TSH gene expressions levels in response to ambient [FT4] concentrations related to epigenetic modifications is one mechanism that accounts for such a perplexing disparity [38]. Another equally important contributory factor to these distressing incongruent clinical outcomes is the differential recovery rates in the spatiotemporal attainment of equilibrium between molecular, cellular, tissue, organ, systemic and biochemical euthyroidism, currently a subject of intense research [7,39]. Clearly, this remains an unresolved scientific issue by the yardstick of our present state of knowledge. But assuming that all these compartments have in fact equilibrated stably and are equally euthyroid after a finite span of time, any residual dysthyroid symptoms imply that the state of euthyroidism is not closely aligned to the patient’s personal homeostatic set point. It is this area of treatment-outcome incongruence that we specifically develop our theory to exact an effective remedy.

The observation that most normal people have tightly regulated, uniquely paired [FT4] and [TSH] that remain relatively constant across time implied that the normal intra-individual euthyroid ranges of [FT4] and [TSH] must be significantly narrower than the population reference ranges and suggest that every human being has an individualized ‘set point’ dictated by the HPT axis as the healthy euthyroid point around which physiological oscillatory variations may occur [1,4]. Thus, the diurnal variations in [FT4] and [TSH] can be visualized as stable miniature hormonal oscillations around the physiological set point analogous to a ball rolling back and forth in a conical cup where the bottom of the cup is the ‘center of gravity’ representing the stable euthyroid set point. In fact, the proper operation of negative feedback loops in metabolic physiology necessitates homeostatic set points that the biological system inherently possesses and targets towards for the maintenance of functions critical to survival of the organism. Hence, though this set point approach to manage thyroid patients seems to be a relatively unsettled concept, it is based on physiological principles firmly grounded in the biomedical literature on various aspects of endocrine regulatory feedback loops [9,11,12].

Our mathematical model of the set point provides credible evidence that this should occur at the position corresponding to the point of maximum curvature on the negative exponential HP curve. This location offers the greatest efficiency for ultra-fine sensitivity of the thyrotrophs to calibrate their [TSH] response robustly to any changes in [FT4]. Remarkably, the second derivative of the curvature function shows a natural tendency for the thyroid secretion of [FT4] to hover tightly in the region encompassing the neighborhood of this set point, and optimal control analysis independently confirmed the high stability of the set point based on maximal loop gain with respect to [TSH] over this spot. Sensitivity analysis revealed that the set point computation based on the parameters S and φ is reasonably precise with appreciable estimation errors only when [FT4] and [TSH] are distant from the normal ‘euthyroid’ ranges (ie. < 3 pmol/L and 0.5 mU/L respectively), thus guaranteeing that predicted euthyroid set points occurring within the reference ranges are likely to be accurate. Thus, for all intent and purposes, our model should yield predicted HPT set points that are sufficiently robust and reliable for routine clinical applications.

To the best of our knowledge, our mathematical theory of the HPT set point is novel and radical, being the very first of its kind ever to have been proposed in the thyroidology literature as there are no parallel conjectures forwarded or published by anyone else to date comparable with ours. The case examples provided cogent proof by directly confirming the observed human data against the matched euthyroid set points as predicted by our theoretical calculations. Indeed, the difference between the patients’ presumed set point [FT4] and the estimated set point [FT4] varied from as low as 0% to about 16% while that for [TSH] varied between 0% to about 38%. In contrast, given the wide population normal ranges in TFT, our present clinical practice can grossly under-estimate or over-estimate the true set point by as much as about 80% and 400% for [FT4] and [TSH] respectively depending on the circumstances. This probably accounts for the commonly observed phenomenon of numerous people who still complain of persistent dysthyroid symptomatology even though they have TFT values falling within the normal ranges.

In conclusion, we have developed a rigorous theoretical framework for computation of the HPT axis set point of any individual which can be used to assist clinicians in the treatment of thyroid patients. This is a revolutionary step forward by allowing clinicians to more precisely target their doses of thyroid medications so as to attain TFTs that fall within the set point region predicted by the theory. Obstacles that have hindered widespread adoption of mathematical calculations among healthcare professionals still abound. But such barriers are gradually eased with the increasing acceptance of computer technology among medical doctors in the modern era. While these calculations may seem cumbersome, we have patented a computerized algorithm currently developed into software (Thyroid-SPOT: Set Point Optimization and Targeting) to greatly facilitate the euthyroid set point determination [40]. We envisage that this computer program can be readily implemented and applied in the clinic and hospital settings for optimal outcomes that many with thyroid disorders will stand to benefit in the age of personalized medicine.

## Limitations

We recognize the lack of a randomized clinical trial to prove the superiority of such a set point-targeted approach over the standard ‘normal range guided’ treatment. A prospective clinical trial using the set point theory as developed above can be employed in future to confirm if patients randomized to a set point-centric approach will have better clinical outcomes compared to those treated using the standard conventional approach. As well, because the construction of an accurate HP curve requires multiple TFT data points, it means that it could take at least 3–6 months before the set point can be estimated. Using only 2–3 paired [FT4]-[TSH] data points obtained at the initial phase of treatment to deduce the set point is thus unlikely to generate an accurate prediction due to possible confounding hysteresis factors likely to operate in the early stages of treatment of either severe hyperthyroidism or hypothyroidism.

## Competing interests

Exploit Technologies Pte Ltd (ETPL), A*STAR’s tech-transfer arm, has filed a patent on the HPT axis set point algorithm that has been developed into a computer program (Thyroid-SPOT software) and MKSL and SLG are both listed as two of three co-inventors. The patent is successfully granted in Singapore.

## Authors’ contributions

SLG and MKSL jointly developed the mathematical model, tested the performance of the model on the clinical data and participated in the analysis of results, optimization and manuscript preparation. Both authors read and approved the final manuscript.

## Authors’ information

Melvin Khee-Shing Leow is a Senior Consultant Endocrinologist in Tan Tock Seng Hospital, Clinical Associate Professor in National University of Singapore, Adjunct Associate Professor in Duke-NUS Graduate Medical School, and Clinician Scientist in Brenner Centre for Molecular Medicine, Singapore.

Simon L. Goede is an Electronic Engineer in Oterlekerweg 4, 1841 GP Stompetoren, The Netherlands.
